# Could Google Trends Be Used to Predict Methamphetamine-Related Crime? An Analysis of Search Volume Data in Switzerland, Germany, and Austria

**DOI:** 10.1371/journal.pone.0166566

**Published:** 2016-11-30

**Authors:** Alex Gamma, Roman Schleifer, Wolfgang Weinmann, Anna Buadze, Michael Liebrenz

**Affiliations:** 1 Department of Forensic Psychiatry, Institute of Forensic Medicine, University of Bern, Bern, Switzerland; 2 Department of Forensic Toxicology and Chemistry, Institute of Forensic Medicine, University of Bern, Bern, Switzerland; 3 Department of Psychiatry, Psychotherapy and Psychosomatics, Psychiatric Hospital, University of Zürich, Zürich, Switzerland; New York City Department of Health and Mental Hygiene, UNITED STATES

## Abstract

**Objective:**

To compare the time trends of Google search interest in methamphetamine and criminal offences related to this drug.

**Methods:**

Google Trends data for the search term "meth" was compared to methamphetamine-related crime statistics (incl. use, possession, and dealing) in Switzerland, Germany, and Austria for the years 2004–2016. Google data was availably monthly. Crime data was available yearly, and monthly values were imputed.

**Results:**

On the country level, internet search trends for "meth" roughly paralleled relevant criminal activity. State-level data, which was available for Austria, showed more heterogeneity. Cross-correlations for yearly data almost always peaked at a lag time of 0 and coefficients were mostly between 0.7 and 1.0 on the country level, and between 0.5 to 1.0 on the state level. Monthly cross-correlations based on imputed values were substantially lower, ranging from 0 to 0.6.

**Conclusions:**

These results encourage the further evaluation by law enforcement authorities of Google search activity as a possible predictor of methamphetamine-related crime. However, several limitations, in particular the crude temporal resolution of available crime data, precluded a detailed assessment of the relationship between internet search trends and the development of methamphetamine-related crime in central Europe.

## Introduction

Methamphetamine is a stimulant drug with strong abuse potential. It acts on the monoamine neurotransmitter system of the brain and increases extracellular concentrations of dopamine, norepinephrine and serotonin [1]. At higher doses it produces feelings of euphoria, extreme wakefulness, rapid flow of ideas, feelings of increased capacity and energy, garrulousness, talkativeness and intense sexual arousal [2]. Methamphetamine can be ingested, smoked, snorted, dissolved in water or alcohol and injected [3]. It is sold as powder, crystals or pressed into tablets. The crystalline form consists of methamphetamine hydrochlorine and is known as "crystal meth" or just "crystal".

Potential adverse effects include dependence, neurocognitive deficits, mental health problems, and cardiovascular, dental, dermatological and sexual health problems[4]. Chronic methamphetamine use may also result in structural and functional brain deficits[5]. Several studies indicate that consumption is furthermore associated with violent, (sexual) risk and criminal behavior [6–8] and methamphetamine use predicts general recidivism among offenders [9].

Methamphetamine is one of the most popular illicit drugs in the world, especially in East and South-East Asia [10], North America [11], and in the Czech Republic and Slovakia [12–15].

In the rest of Europe, methamphetamine use was relatively rare before 2008 [13], but has since grown significantly [16–19], leading to an increase of meth-related offences as well as police response. In Germany, it is particular the states bordering on the Czech republic, Bavaria and Saxony, that have seen the largest methamphetamine problem [20,21].

"Google Trends" is a web service offered by Google to track the popularity of terms entered in its search engine. It delivers data on the frequencies of search terms broken down by geographical location and time. The data are relativized to the total search volume for that term in the specified region at the specified time. Thus, the data are not absolute, but normalized, numbers.

Research using Google Trends and other user-generated internet data has experienced a dramatic rise in recent years [22]. Considerable work has focused on tracking acute diseases, the best-known example being Google Flu Trends (GFT), a service established by Google in 2008 to predict spatiotemporal patterns of influenza activity [23].

Another promising use of internet data is as a tool in behavioral medicine to survey health-related online behaviors that may yield actionable insights for policymakers [24]. Such data is especially useful where traditional systems of surveillance are not available. For example, Ayers et al have demonstrated using Google search trends that the popularity of e-cigarettes surpassed that of other smoking alternatives or cessation devices at a time when academic interest was still focused on the latter [25].

Internet search data offers a unique set of advantages and challenges. It is big, publicly available, and real-time. It is not subject to many of the social and psychological biases created in experimental or survey settings (e.g. social desirability, experimenter bias etc.). In general, however, the context of online behaviors is unknown and their motives remain difficult to determine, despite attempts to address the issue by including search terms indicating possible motives. For example, Ayers et al have used terms such as 'buy' and 'shop' or 'health' and 'risk to assess motives for searching for e-cigarettes on the internet [26].

The problem of motives is part of the general problem of validity of internet data. Not knowing the meaning of an online behavior means not knowing what exactly it tracks or what it might predict. For example, do search trends for influenza reflect actual prevalence of influenza? Despite the generally good performance of GFT, a number of severe prediction failures [27,28] demonstrated that this is not necessarily the case. Search queries can be confounded by various influences, among them shifts in public and media attention to the target phenomenon that do not reflect actual changes in its prevalence [28]. As a result, internet surveillance does not always outperform or even add value to traditional information such as CDC-issued predictions of influenza prevalence. There is therefore a need to combine novel digital as well as traditional analog sources of surveillance [27,29].

In the current study we compare interest in Google searches for methamphetamine with relevant drug crime statistics in Switzerland, Germany and Austria, for the years since 2004. The aim is to evaluate the temporal relationship of these two trends. We focus on the question of "raw predictability" of criminal activity by search trends without making assumptions about user motives, thereby alleviating concerns about validity.

## Methods

### Google Trends Data

An overview of search parameters used with Google Trends is given in Table 1. Two searches were run, one for the term “meth” (“search 1”) and one for “crystal meth” and variants (“search 2”). Search term variations for crystal meth were chosen based on Google Trends' result category "most frequent searches" for “crystal meth” and were required to contain a variant of the descriptor "crystal". The four most frequent variants were included in search 2. Note that the term "meth" in search 1 will include results from those more specific terms in search 2 that contain "meth" as a sub-term.

**Table 1:** Search parameters used for Google Trends.

Google data source	Google Trends
Type of Search	Web Search
Query Category	All Categories
Date of access	14.02.2016
Time period	Jan 2004 –Feb 2016
Time division	Month
*Search terms*:	
Search 1	“meth”
Search 2	"crystal meth" + "cristal meth" + "crystel meth" + "chrystal"

Data were retrieved on February 14, 2016, for the period from January 2004 to February 2016 at monthly intervals. We searched the countries Switzerland, Germany, Austria, Czech Republic, and Poland, as well as their states, using the “web search” service and including “all categories”.

Due to often low or zero search volumes for search 2, we present results only for search 1 (“meth”).

### Crime Statistics

An overview of available crime statistics is given in Table 2.

**Table 2:** Available crime statistics related to methamphetamine use.

Switzerland	Time period	Germany	Time period	Austria	Time period
*Offences*^*a*^^*c*^		*Police operations*^*a*^^*c*^		*Offences*^*b*^^*c*^	
N total meth-related offences	1998–2014	N seizes^d^	2006–2014	N illegal possession/dealing	2004–2014
N use	2009–2014	Amount [kg] seized^d^	2006–2014		
N possession	2009–2014	N illegal drug labs busted^e^	2005–2014		
N dealing	2009–2014	*Users*^*a*^^*c*^			
N manufacture	2009–2014	N first users^d^	2006–2014		
N import/transit/export	2009–2014				
*For all of the above categories*:					
N offenders (2009–2014)	2009–2014				
*Not crime-related*:					
N hospital admissions with ICD-10 diagnosis F15.x (stimulant use)	1998–2014				

^a^Country-level data

^b^State-level data

^c^Yearly data

^d^Concerns only crystal meth; data for other forms of methamphetamine is combined with amphetamine

^e^Not specific to methamphetamine; labs also produce other substances such as amphetamine, MDMA, and GHB

N = number

Crime statistics were retrieved from the Swiss Federal Statistical Office [30] in Switzerland, from the Federal Criminal Police Office (“Bundeskriminalamt”, BKA) in Germany [21], and from the Federal Office of Criminal Investigation (“Bundeskriminalamt”) in Austria [31]. In addition, we obtained data on hospitalizations in Switzerland related to ICD 10: F15 [30].

Unfortunately, state-level data was only available for Austria.

### Statistics

Search volumes for the different variants of "crystal meth" were summed up. Due to very low or zero search volume for crystal meth in most states, only the search term "meth" was included in further analyses.

Search volumes for "meth" and crime statistics were plotted against time for the different countries and states within them. To assess whether search interest preceded or trailed methamphetamine-related offences, we calculated cross-correlations between search volume for "meth" and every crime statistic. As crime data was not available monthly, we report cross-correlations for years, summing up monthly search volumes per year. Depending on the number of years available in the time series, lags up to ± 2 years were computed.

However, using yearly data will overestimate correlations, because the monthly variation is lost. To get a sense of this variation, we imputed monthly values under the constraint that they sum to the corresponding yearly value. Each crime variable was filled up with sets of 12 monthly values each that were sampled randomly from a Dirichlet distribution with alpha parameters set to 1. The Dirichlet distribution embodies the constraint that its values sum to 1. Scaling the resulting sets by the corresponding yearly values guarantees that the samples in the set sum to the yearly value. This procedure was repeated 100 times for each crime variable, each time calculating the cross-correlation with search volume for "meth". The median and interquartile range of these cross-correlations are reported here. All analyses were done and Stata 14.1 for Mac [32] and in R 3.2.4 for Mac [33].

## Results

### Time course of search volume and crime statistics

**Fig 1:**
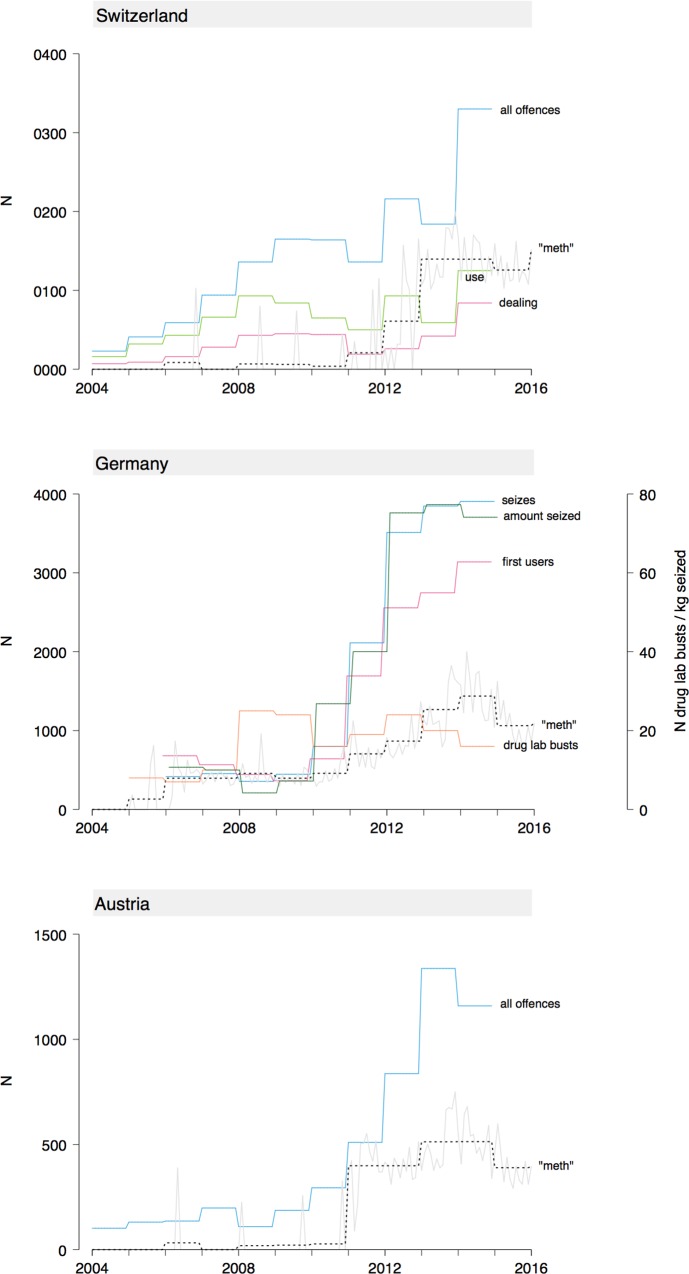
Internet search activity and crime statistics related to methamphetamine in Switzerland, Germany, and Austria. Search volume data is available monthly, crime statistics yearly. The category "All offences" in Switzerland includes use, possession, dealing, manufacture, and trafficking. In Austria, it includes possession and dealing. Number of seizes, amount seized and number of first users in Germany refer to crystal meth only, since other forms of the drug are lumped together with amphetamine in the official statistics. The corresponding curves have been slightly horizontally offset from each other for better visibility. Search volume for the term "meth" is a relative number, whose scale is not informative and therefore not shown. For ease of visual comparison, it has been rescaled in each graph to peak at half the maximum of the y-axis. The dashed black line represents the averaged monthly search volume within a year to make values more easily comparable to crime statistics.

The time course of Google search activity and crime statistics related to methamphetamine are shown in Fig 1.

Overall, criminal offences and police activity related to methamphetamine rose steeply between 2010 and 2014 in German-speaking European countries. Google search interest followed the same general pattern.

Methamphetamine-related offences in Switzerland (including use, possession, dealing, production and trafficking) have slightly increased since 2004, and more sharply after about 2012. Hospitalizations have steadily risen since 2004. We also looked at separate subcategories of offences (e.g. use, possession, dealing, smuggling), but, failing to find any striking deviations from the total category, decided not to examine cross-correlations for these data.

In Germany, the number of first users of methamphetamine, the number of drug seizes and the amount seized all started to increase significantly in 2010/2011.

**Fig 2:**
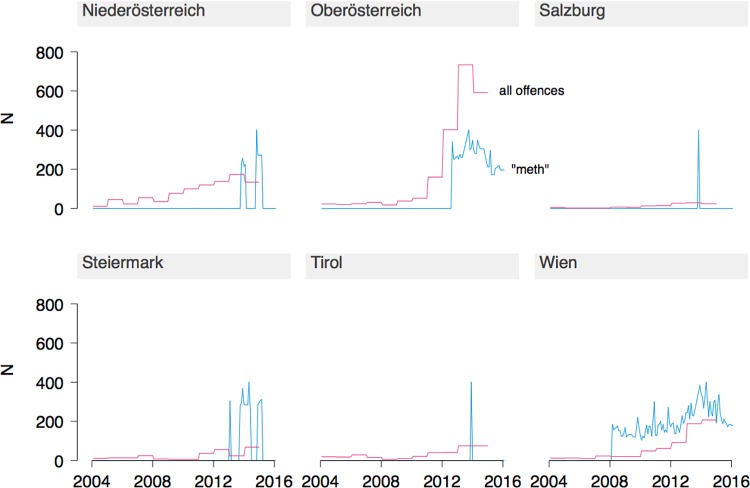
Time course of criminal offences and Google search volume related to methamphetamine in states of Austria. "All offences" includes possession and dealing of the drug. Search volume is a relative number and has been re-scaled to peak at half the maximum of the y-axis.

State-level crime data was available for Austria. Similar to Germany, methamphetamine-related offences (use, possession, and dealing) started to increase significantly around 2010, however only in certain states. The rise was most pronounced in "Oberösterreich" (which borders on the Czech Republic), and less so in "Wien" (Vienna) and "Niederösterreich" (which borders on the Czech and Slovak Republics). Search volume and criminal offences by state are shown in Fig 2.

### Cross-correlations between search volume and crime statistics

**Fig 3:**
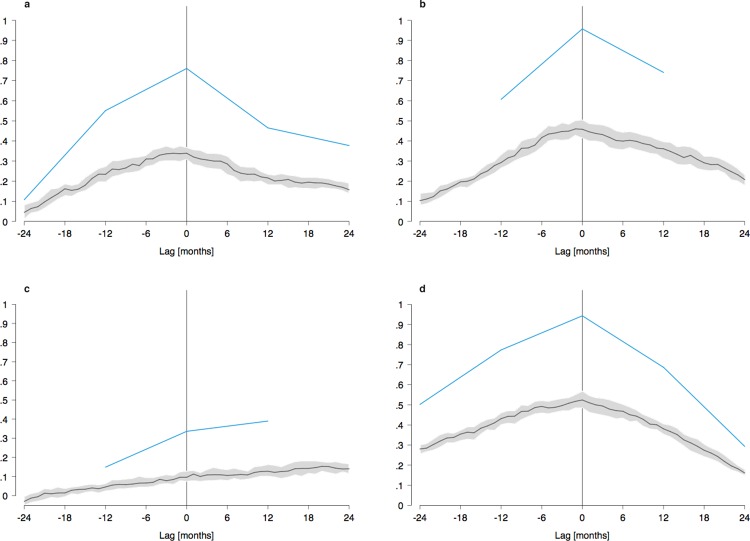
Cross-correlation of Google search volume and criminal offences related to methamphetamine. The blue curve shows cross-correlations based on yearly values. The gray line and shaded area represent the median and interquartile range of monthly correlations based on imputed data. a Total offences in Switzerland. b Number of first methamphetamine users in Germany. c Number of drug lab busts in Germany. d Total offences in Austria.

Cross-correlations based on yearly values were almost uniformly highest at a lag of 0 years as compared to lags of ±1 or ±2 years. On the country-level, these peaks showed correlations between 0.7 and 1.0, with the exception of the number of drug lab busts in Germany, which peaked at about 0.4 at a lag of 1 year (Fig 3). In the states of Austria, yearly cross-correlations varied between 0.5 and 1.0, again mostly peaking at a lag of 0 years.

Using imputed monthly values for the crime data substantially lowered peak correlations with search volume. On the country level, reductions were around 0.5, yielding peak values between 0.3 and 0.5 (Fig 3). In the states of Austria, the reduction was between 0.3 and 0.5, resulting in peak monthly cross-correlations between 0 and 0.6.

Interquartile ranges around peak monthly cross-correlations ranged between .07 and .10 (Fig 3). Not surprisingly, the shapes of the monthly cross-correlation patterns generally followed those of the corresponding yearly patterns.

## Discussion

We found that time trends in Google search interest for methamphetamine in three middle European countries roughly paralleled methamphetamine-related offences and other crime statistics. This was not a uniform finding, however, especially when looking at the state level. Austrian states showed a variety of patterns, with search activity leading (Wien) or lagging behind (Oberösterreich) drug offences, or showing no obvious relationship. In Germany, the number of drug lab busts also showed no apparent association with search volume, one likely reason being that these laboratories produce various drugs, not only methamphetamine.

Nevertheless, the overall parallelism of drug offences and search interest for methamphetamine on the country level encourages the evaluation of Google Trends by legal and law enforcement authorities as a predictive instrument for drug-related crimes. Ideally, such information could contribute to more effective interference by police in drug production and distribution networks. In health care, predictive data could help to steer the deployment of both prevention and harm reduction efforts more efficiently [34]. In cases when geographical information on search trends is available, measures could be targeted to locations where spikes in drug availability are expected [35].

Beyond this general recommendation, further interpretation of our results is limited by the considerable uncertainty in the data. First, we had no monthly crime data to match the monthly search volumes, therefore the data only supported rough yearly cross-correlations. This made it impossible to assess the lag between the two trends on a more fine-grained level.

Second, there was no state-level data for Switzerland and Germany, a fact which might have obscured or artificially generated correlations between search volume and crime statistics on the country level. This can be seen in the case of Austria, where country-level analysis masked substantial state-level heterogeneity in cross-correlations. Third, the sparseness of data points in some Austrian states precluded the detection of potential correlations. Fourth, the time-series is not very long. Fifth, there is likely to be variation in the dating of offences and police operations, generating noise in lag or lead times relative to internet search activity. Sixth, we had no data on non-crime-related variation in police activity, as for example, when changes in police activity merely reflect changes in political climate.

Finally, and more generally, we do not know what variety of motives underlie the searches we identified: an intent to buy the drug, the curiosity of a potential prospective user, the concern of a parent, or the research of a journalist. While the fact of similar trajectories supports some connection to local availability and use of the drug, we do not know the direction of causality: do internet users search for methamphetamine as a reaction to local media reporting increased drug availability or do they hear about the drug being increasingly offered in clubs and then google it? In the former case, the search volume generated would probably be late with respect to police awareness of the drug. In the latter case, the search activity could precede peaks in drug-related illegal activity and actually be of predictive value to law enforcement. Here, only a finer temporal resolution of crime data matching that of search volume data could provide an answer. Nevertheless, it is probably safe to assume that both mechanisms, and maybe additional ones, are at play to some extent.

In conclusion, the current study is part of an emerging field of internet research whose primary measures are online behaviors which need to be better understood and validated. Our findings represent a small contribution towards that goal by supporting the possibility that internet search trends could predict drug-related criminal activity.
